# Gold Nanoparticles Impinge on Nucleoli and the Stress Response in MCF7 Breast Cancer Cells

**DOI:** 10.5772/62337

**Published:** 2016-01-01

**Authors:** Mohamed Kodiha, Hicham Mahboubi, Dusica Maysinger, Ursula Stochaj

**Affiliations:** 1 Department of Physiology, McGill University, Montreal, QC, Canada; 2 Department of Pharmacology & Therapeutics, McGill University, Montreal, QC, Canada

**Keywords:** Gold Nanoparticles, Gold Nanoflowers, Nucleolus, Stress Response, Heat Shock Protein 70

## Abstract

Cancer cells can take up gold nanoparticles of different morphologies. These particles interact with the plasma membrane and often travel to intracellular organelles. Among organelles, the nucleus is especially susceptible to the damage that is inflicted by gold nanoparticles. Located inside the nucleus, nucleoli are specialized compartments that transcribe ribosomal RNA genes, produce ribosomes and function as cellular stress sensors. Nucleoli are particularly prone to gold nanoparticle-induced injury. As such, small spherical gold nanoparticles and gold nanoflowers interfere with the transcription of ribosomal DNA. However, the underlying mechanisms are not fully understood. In this study, we examined the effects of gold nanoparticles on nucleolar proteins that are critical to ribosome biogenesis and other cellular functions. We show that B23/nucleophosmin, a nucleolar protein that is tightly linked to cancer, is significantly affected by gold nanoparticles. Furthermore, gold nanoparticles impinge on the cellular stress response, as they reduce the abundance of the molecular chaperone hsp70 and O-GlcNAc modified proteins in the nucleus and nucleoli. Together, our studies set the stage for the development of nanomedicines that target the nucleolus to eradicate proliferating cancer cells.

## 1. Introduction

Breast cancer continues to be the most frequent form of cancer in women worldwide [1]. This disease burden is heightened by complications that limit the success of current therapies including the heterogenic nature of malignant tumours, crosstalk between different cell signalling systems and the development of drug resistance [2–5]. The genomic diversity between and within malignant tumours makes it particularly challenging to develop new therapeutic tools and protocols that benefit a large cohort of cancer patients.

To increase the efficiency of current cancer treatments, our research focuses on two essential intrinsic cancer cell support systems: the nucleolus and molecular chaperones. Nucleoli reside inside the nucleus where they assemble ribosomes, regulate cell cycle progression, telomerase activity and apoptosis [6, 7]. Furthermore, nucleoli serve as stress sensors [7], and their proper organization and function depends on molecular chaperones [8]. Nucleoli are crucial for tumour biology because proliferating cancer cells rely on elevated ribosome production. The cancer-associated rise in ribosome biogenesis causes nucleolar hypertrophy, a hallmark of many tumour cells [9–12]. Notably, the degree of nucleolar hypertrophy correlates with tumour malignancy, especially for breast cancer, and is inversely related to patient survival [10, 13]. Relevant to our studies, nucleolar organization and function are sensitive to stress including heat shock [14, 15].

Mammalian nucleoli are organized into three major subcompartments. They can be divided into fibrillar centres, dense fibrillar components and granular components. As these subcompartments participate in different steps of ribosome biogenesis (reviewed in [16]), their proper organization is critical for nucleolar functions. We analysed proteins that are present in different nucleolar subcompartments and directly linked to cancer. Specifically, B23/nucleophosmin (here referred to as B23) and nucleostemin are abundant in the granular component. RPA194, the largest and catalytic core subunit of RNA polymerase I, resides in the fibrillar component, and the multifunctional protein nucleolin associates with both the dense fibrillar and granular components ([17, 18] and references therein).

B23/nucleophosmin is a multitasking protein that can function as a tumour suppressor or proto-oncogene. Moreover, mutant B23 is a major contributor to acute myeloid leukaemias [19–21]. Interestingly, B23 also acts as a molecular chaperone (reviewed in [6, 22]). Nucleolin is of particular interest to tumour biology because this protein is not only present in nucleoli; it is also located on the surface of many cancer cells [23–25].

RNA-polymerase I transcribes the 45S precursor of 28S, 18S and 5.8S rRNA [26]. Several small compounds that inhibit RNA-polymerase I lead to the cleavage of RPA194 [27, 28]. This cleavage can limit the transcription of rRNA genes in nucleoli, thereby reducing ribosome production. Finally, the GTP-binding protein nucleostemin is believed to protect genome integrity (reviewed in [29]). All of these proteins play a role in tumour biology and, thus, represent potential therapeutic targets for cancer treatment.

As a stress sensor, the nucleolus is sensitive to changes in cell physiology [7]. Molecular chaperones, in particular members of the hsp70 family, help maintain and restore the organization and function of nucleoli, especially after exposure to stress [6, 8, 15, 30, 31]. Hsp70s are highly abundant in breast cancer cells [32]. They increase tumour cell survival through multiple pathways, including cell signalling and the prevention of apoptosis [32, 33].

Posttranslational modifications are also part of the cellular stress response. For example, serine and threonine residues can be modified by O-linked N-acetylglucosamine (O-GlcNAcylation), which is common for transcription factors and nuclear pore proteins [34]. Transient O-GlcNAcylation promotes the survival of acute stress, whereas reduced O-GlcNAcylation correlates with diminished stress-induced hsp70 synthesis [35]. Notably, breast cancer cell lines have high levels of O-GlcNAcylation [36]. This determines cancer cell metabolism and survival [36]. Molecular chaperones and O-GlcNAcylation also affect cellular proteostasis [37], and chaperones such as hsp70 undergo O-GlcNAcylation [38].

Work by us and others identified nucleoli as potential targets for tumour cell killing [39, 40]. Various pharmacological compounds alter the activity and organization of nucleoli [17, 41, 42]. However, these agents frequently have severe side effects. Treatments that are directed more selectively towards cancer cells could overcome some of these obstacles. Gold nanoparticles (GNPs) have the potential to achieve this goal. They are inert, biocompatible and excellent tools for cancer research and therapy. GNPs are easy to detect and ideal for inducing photothermal killing in a localized fashion [43–46].

GNPs are available in different sizes and morphologies. These particle properties determine GNP cellular uptake, damage and ultimately cancer cell death [47–49]. Moreover, due to the enhanced permeability and retention properties of the tumour vasculature, some GNPs accumulate in tumour tissues [50]. Such an accumulation is determined by the physicochemical characteristics of the particles. For example, permeation into the tumour tissue is diminished for large GNPs [50]. The cellular damage afflicted by spherical and rod-shaped GNPs or nanoclusters has been examined extensively (reviewed in [51, 52]). By contrast, much less is known about the biological impact of branched GNPs such as gold nanostars [53] or nano-urchins [54]. These GNPs display a large surface/volume ratio and can be used for photothermal ablation with near-infrared light [55]. The damage to the neighbouring healthy tissue exposed to near-infrared light is minimal and, therefore, presents an attractive therapeutic modality. Future treatments that rely on GNPs, which associate preferentially with cancer cells, could further reduce treatment side effects by restricting the distribution of GNPs.

We have previously shown that small gold nanospheres (∼ 15 nm in diameter) and gold nanoflowers damage the nucleus and impair the transcription of ribosomal RNA genes (rDNA), the first step in the production of ribosomal subunits [49]. These GNPs, especially when combined with mild heat stress, alter the viability and proliferation of MCF7 breast cancer cells. To obtain a better understanding of the underlying mechanisms, we focused on nucleolar proteins and components of the stress response that are implicated in nucleolar functions. Our research identified novel components that respond to the treatment with small gold nanospheres and nanoflowers in breast cancer cells.

## 2. Experimental Procedures

### 2.1 Reagents

**Figure A1.**
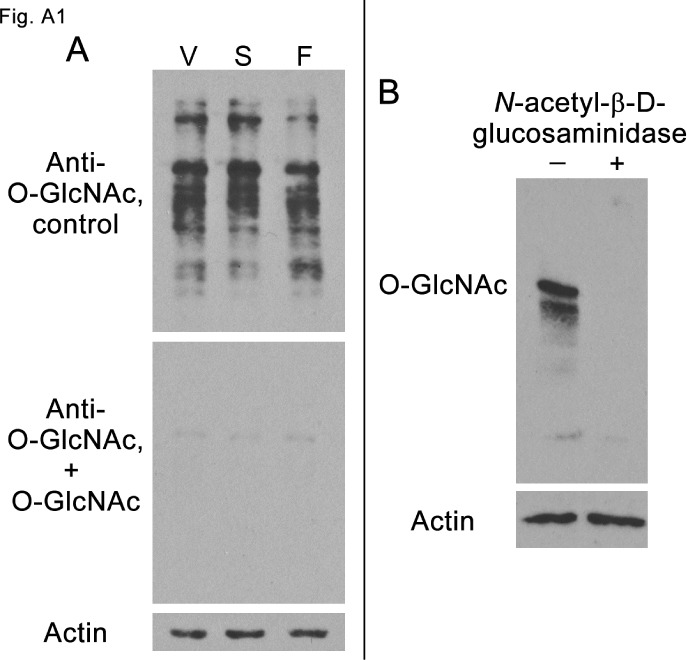
Specificity of antibodies against O-GlcNAc modifications. (A) Crude extracts were prepared for MCF7 cells incubated overnight with vehicle (V), small gold nanoparticles (S) or gold nanoflowers (F). Identical aliquots were analysed by Western blotting with anti-O-GlcNAc antibodies in the absence (top panel) or presence of 100 mM O-GlcNAc (middle). Actin is shown as loading control in the bottom panel. (B) MCF7 proteins were solubilized in SDS and incubated in the absence or presence of 100 U/ml *N*-acetyl-β-D-glucosaminidase, as described [57]. Actin shows comparable loading for both samples.

Antibodies against the following proteins were used for immunofluorescence and Western blotting: B23/nucleophosmin (Cell Signalling, #3542), nucleolin (Santa Cruz, sc-13057), nucleostemin (R&D Systems, AF1638), RPA194 (Santa Cruz, sc-48385), mab414 (BabCo), hsp70 (Enzo Life Sciences, SPA-812), O-GlcNAc (antibody RL2, ABR, MA1-072), phosphorylated (Thr172) and total AMPKα (Cell Signalling, #2535, #2532). Figure A1 examines the specificity of antibody RL2 for the O-GlcNAc epitope. The addition of free O-GlcNAc (Sigma) during Western blotting efficiently reduced the enhanced chemiluminescence signals (Figure A1, part A). Epitopes recognized by RL2 were not removed by treatment with peptide *N*-glycanase F [56]. However, as shown in Figure A1 (part B), RL2 binding was profoundly diminished after incubation with *N*-acetyl-β-D-glucosaminidase, an enzyme that removes O-GlcNAc modifications [57].

### 2.2 Gold nanoparticles

**Figure A2.**
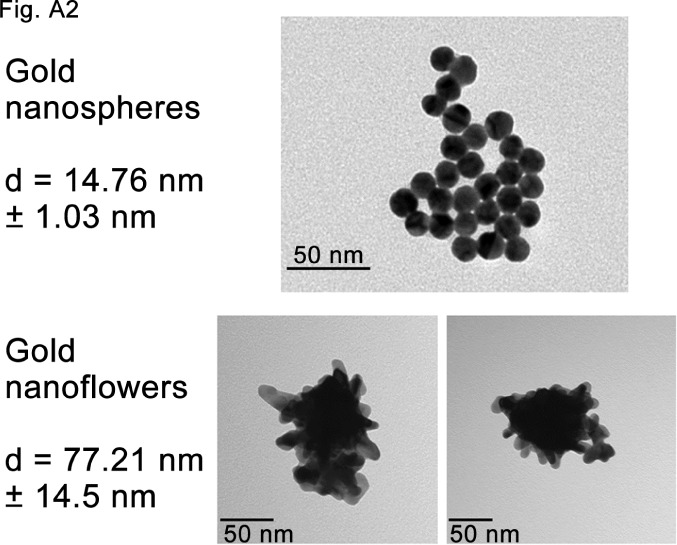
Characterization of gold nanoparticles. Small gold nanospheres and gold nanoflowers used in this study were visualized by transmission electron microscopy following our published procedures [49].

Cetyltrimethylammonium bromide (CTAB)-coated spherical gold nanoparticles were synthesized and characterized as published ([49], Figure A2). Gold nanoflowers were prepared using a modified version of a previously described protocol [58]. In brief, 300 ml pure ethylene glycol was heated to 40°C in a water bath. Under mild stirring, aqueous HAuCl_4_ (1% w/w, 3.0 ml) and freshly prepared triethanolamine solution (2 M, 6.0 ml) were added. The reaction was allowed to run for 1 hour. Gold nanoparticles were then collected by centrifugation (7,000 rpm, 20 min), washed once with deionized water and redispersed in water. The particles were PEGylated by an overnight incubation with α-methoxy-ω-mercapto poly(ethylene glycol) (MeO-PEG-SH; 5,000 g/mol; 0.1 mM), concentrated by centrifugation and washed with water. The final concentration was determined by inductively coupled plasma mass spectroscopy (ICP-MS) with a NexIon 300x (Perkin Elmer) instrument.

For the current studies, the size of the gold core was 14.76 ± 1.03 nm for the gold nanospheres (here referred to as “small” gold nanospheres) and 77.21 ± 14.5 nm for the gold nanoflowers. The visualization of gold nanoflowers by transmission electron microscopy and 3D reconstructions with Imaris 8.1 followed our published methods [49].

### 2.3 Cell culture and heat exposure

MCF7 cells were maintained and treated with gold nanoparticles, as published [49]. In brief, the cells were incubated overnight with 19.7 μg/ml gold nanoparticles or vehicle. After 1 hour hyperthermia at 43°C, the samples were returned to 37°C for a 2-hour recovery period.

### 2.4 Evaluation of nucleolar proteins

Four essential nucleolar marker proteins were evaluated: B23/nucleophosmin, nucleolin, RPA194, and nucleostemin [17]. In addition, signals were measured for hsp70 and O-GlcNAcylation. At least 35 cells and 32 nucleoli were assessed for each experiment and data point. All secondary antibodies were affinity purified and pre-adsorbed to mammalian proteins. As described [18, 59], non-specific binding of secondary antibodies was negligible under these conditions.

Earlier, we established the methods to delimit nucleoli and quantify fluorescent signals in nucleoli and the nucleus [14, 15, 17, 18, 60]. In brief, the software uses multiple criteria to delimit the nucleolar compartment. These criteria include the minimum and maximum width of the compartment, together with the difference in fluorescence intensities between nucleoli and the surrounding nucleoplasm. Each set of experiments used an established marker that identified nucleoli as “dark holes” or “bright holes”. Upon correction of background pixel intensities, the fluorescence signals were measured in the regions demarcated as nucleoli. We described previously the detailed protocols that were applied here [14, 18].

### 2.5 Western blotting

Western blotting and enhanced chemiluminescence were performed according to our published methods [49].

### 2.6 Metabolic activity

A standard MTT (thiazolyl blue tetrazolium bromide) assay was used to evaluate the toxicity of gold nanoparticles [49].

### 2.7 Statistics

Significant differences were identified by One-way ANOVA combined with Bonferroni correction. Differences were considered significant for *p*<0.05.

## 3. Results and Discussion

### 3.1 Gold nanoparticles affect nucleolar subcompartments

**Figure 1.**
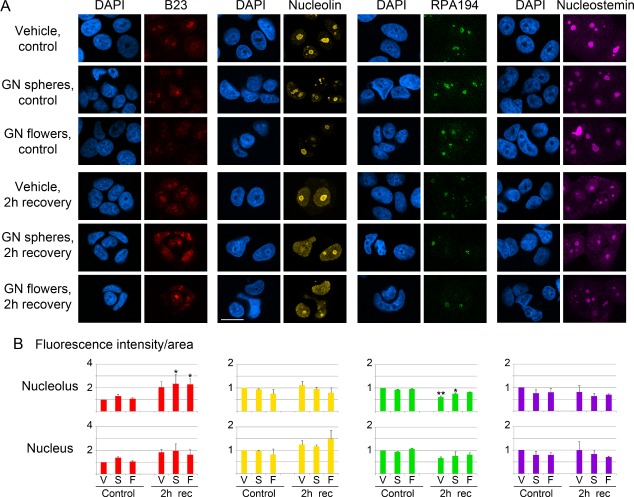
Effect of gold nanoparticles on the nucleolar proteins B23, nucleolin, RPA194 and nucleostemin. (A) MCF7 cells were incubated overnight with vehicle (V), small gold nanospheres (GN spheres, S) or gold nanoflowers (GN flowers, F). In parallel experiments, MCF7 cells were exposed to hyperthermia, followed by a 2-hour recovery period (2h rec), as detailed in the experimental procedures section. Nucleolar proteins were detected by indirect immunofluorescence. The size bar is 20 μm. (B) The fluorescence intensity/area was quantified for nucleoli or the nucleus. The graphs depict averages +SEM for two to four independent experiments. Significant differences were identified with One-way ANOVA and Bonferroni correction. The vehicle control was the reference for all pairwise comparisons; *, *p*<0.05, **p<0.01.

Nucleoli are organized into subcompartments that participate in different biological functions. The proper localization of individual proteins to these subcompartments controls nucleolar activities. As shown in Figure 1, during the recovery from mild hyperthermia B23 redistributed significantly in MCF7 cells treated with small gold nanospheres (average diameter of gold core 14.76 nm) or gold nanoflowers (average diameter of gold core 77.21 nm). The same trend was observed for the nucleus, although the changes were not significant. It is possible that the combination of gold nanoparticles and hyperthermia stimulated B23 nucleolar accumulation through its relocation from other nuclear subcompartments. However, our experiments do not rule out that cytoplasmic B23 also redistributed to the nucleolus.

**Figure 2.**
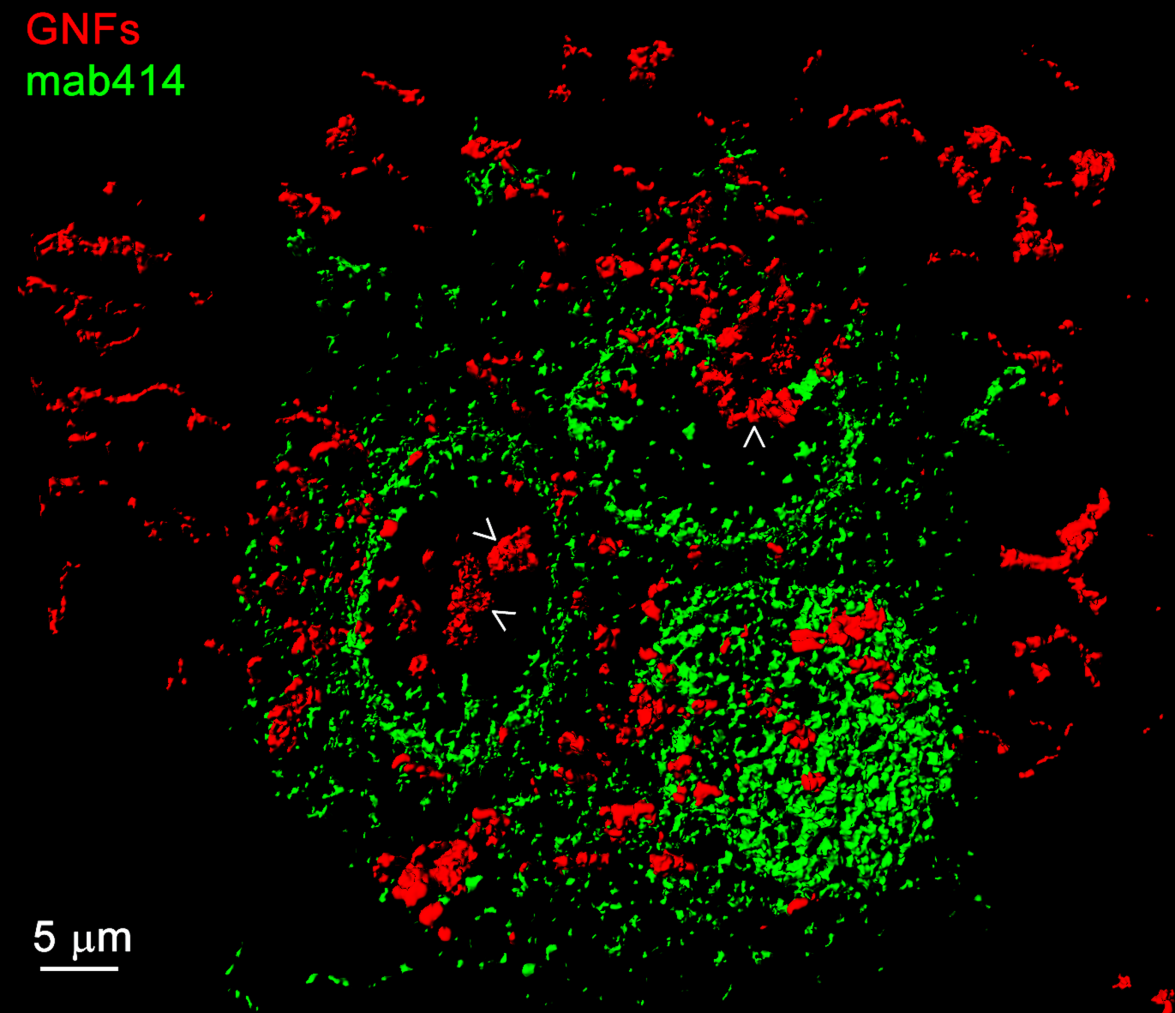
MCF7 cells were incubated overnight with gold nanoflowers (GNFs, red), and nucleoporins were stained with mab414 (green). This monoclonal antibody recognizes nucleoporins that contain FXFG repeats. We performed 3D reconstructions on confocal stacks; arrows point to gold nanoflowers in the nuclear interior. The animation (Movie 1) indicates the intranuclear location of gold nanoflowers. Movie 1 (http://cdn.intechopen.com/public/docs/supplemental/11.avi) - The rotation of the 3D reconstruction in Figure 2 further supports the idea that gold nanoflowers were inside the nucleus.

Since B23 is mostly nuclear in untreated MCF7 cells, our results suggest that gold nanoparticles may be associated with the cell nucleus. This was indeed the case, as small gold nanospheres were present in nuclear fractions [49]. Moreover, gold nanoflowers could be detected inside the nucleus. Thus, the data obtained by nuclear demarcation with mab414, an antibody that binds nucleoporins with FXFG-repeats, suggest that gold nanoflowers were in part located in the nuclear interior (Figure 2, Movie 1).

Nucleolin is a multifunctional protein that is present in different cellular locations and participates in diverse functions (reviewed in [22, 23]). Nucleolin associates predominantly with nucleoli, where it stimulates rDNA transcription and ribosome assembly. In the nucleoplasm, nucleolin inhibits DNA replication, especially under conditions that require DNA repair. In combination with heat shock, the gold nanoflowers increased nucleolin concentrations in the nucleus but these changes were not significant (Figure 1). Together, these data suggest that the intracellular nucleolin distribution is not profoundly affected by GNPs.

Like B23 and nucleolin, RPA194 is a potential target for cancer therapy. For example, the antitumour agent BMH-21 reduces RNA-polymerase I activity [27, 28] and promotes RPA194 degradation. In our experiments, RPA194 relocated significantly during the recovery of vehicle-treated cells (Figure 1). However, this relocation was abolished by gold nanoflowers and reduced for gold nanospheres.

Nucleostemin protects breast cancer cells against DNA damage [61] and stimulates breast cancer growth [62]. In MCF7 cells, GNPs did not elicit significant changes to the nucleolar or nuclear abundance of nucleostemin.

**Figure 3.**
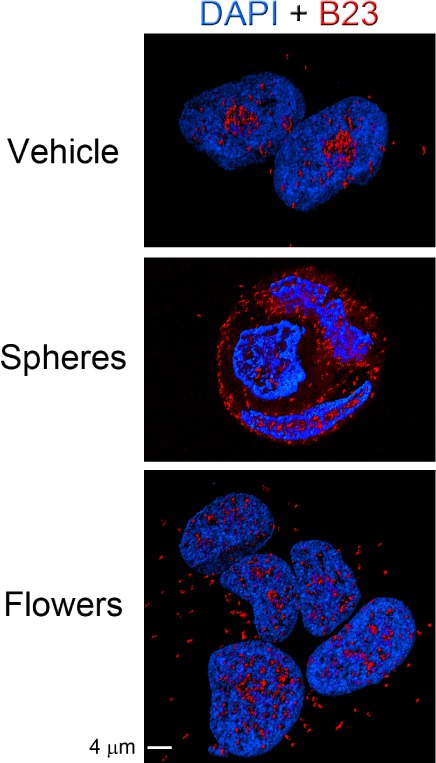
GNPs induce the redistribution of B23 in breast cancer cells. MCF7 cells were incubated at 37°C with vehicle, gold nanospheres or gold nanoflowers, as described for Figure 1. Vehicle-treated cells concentrated B23 in nucleoli, but exposure to GNPs led to B23 relocation. Confocal stacks were used for 3D reconstruction, as published earlier [49]. The size bar is 4 μm. Note that the 3D reconstructions provide information on the subcellular distribution of B23 that goes beyond the nucleolus and nucleus (Figure 1). Specifically, Figure 3 reveals that B23 levels change in the cytoplasm of GNP-treated cells, even without hyperthermia.

Given that GNPs affected B23 localization, we performed confocal microscopy combined with 3D reconstruction to better assess its subcellular distribution, especially in the cytoplasm (Figure 3). B23-containing foci were detected in the nucleoplasm and cytoplasm, and these foci were particularly abundant after incubation with GNPs. Thus, numerous cytoplasmic foci were observed, especially after treatment with small gold nanospheres, supporting the idea that GNPs prompt a marked redistribution of B23.

We have shown earlier that small gold nanospheres and nanoflowers impair *de novo* transcription in nucleoli [49]. Interestingly, during the recovery from heat stress, B23 abundance *increased* in nucleoli and this rise was significant for the cells treated with GNPs (Figure 1). While the physiological relevance of this nucleolar accumulation is presently unknown, it is noteworthy that B23 controls the nucleolar association of DNA base excision repair factors [63, 64]. The hypothesis that GNPs, in combination with mild hyperthermia, induce DNA damage in nucleoli that require repair has to be tested in future studies.

Collectively, our results show that the examined nucleolar proteins responded differently to GNPs. Prominent changes were observed for B23 and RPA194 when GNPs were combined with mild hyperthermia. B23 is a multitasking protein and particularly abundant in the granular component of the nucleolus, where late steps of pre-rRNA processing take place [16]. This indicates that GNPs not only impair *de novo* rDNA transcription [49], but also may compromise the proper processing of ribosomal RNA. Interestingly, B23 interacts with chaperones, including members of the hsp70 family [65].

Unlike B23, the nuclear and nucleolar distribution of other nucleolar proteins was less affected, suggesting that B23 serves as a major indicator of GNP-induced damage, at least in MCF7 cells. Future experiments will have to determine whether these changes are caused by a direct interaction between B23 and GNPs.

### 3.2 Gold nanoparticles alter key components of intrinsic cancer cell support systems

**Figure 4.**
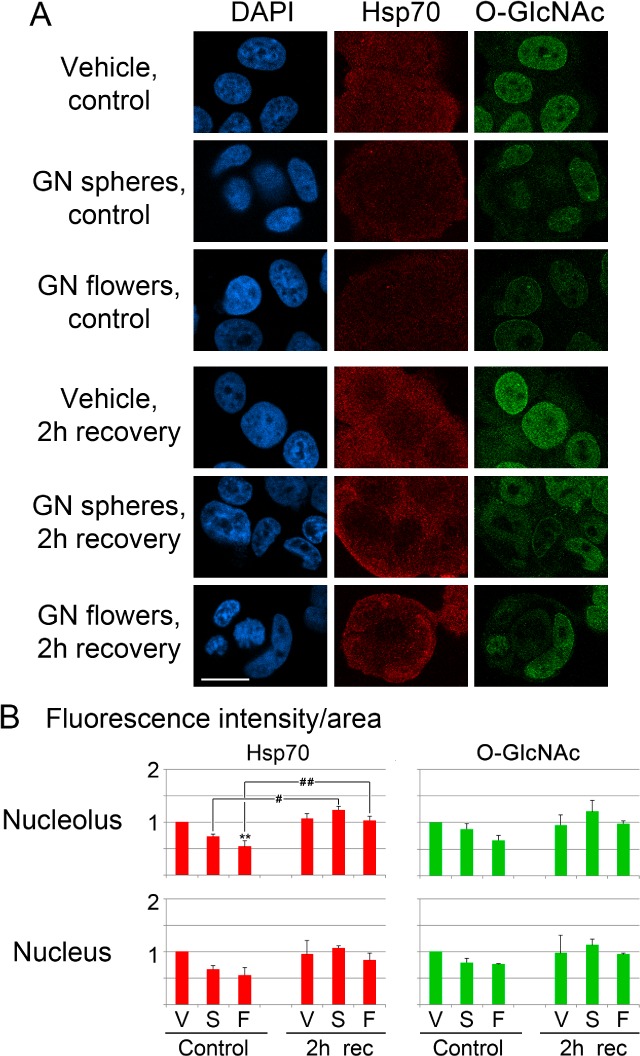
Nuclear hsp70s and O-GlcNAcylation are affected by gold nanoparticles. MCF7 cells were incubated with vehicle or gold nanoparticles, as described for Figure 1. (A) Indirect immunofluorescence detected the inducible chaperone hsp70 or O-GlcNAcylation. (B) Nucleolar and nuclear fluorescence intensities were quantified for at least two independent experiments. The results were normalized to vehicle control samples. Bar graphs depict averages +SEM. Significant differences were identified by One-Way ANOVA, followed by Bonferroni correction. Comparisons were relative to vehicle controls; **, *p*<0.01. Alternatively, all of the samples (controls and 2h recovery) were pairwise compared using One-Way ANOVA/Bonferroni correction; #, *p*<0.05; ##, *p*<0.01.

Molecular chaperones and modification by O-GlcNAcylation are indispensable for proteostasis, especially during stress. Heat shock proteins and O-GlcNAcylation are also particularly important for the survival of tumour cells. Figure 4 examines the impact of GNPs on the stress-inducible chaperone hsp70 and on O-GlcNAc-modified proteins. Small gold nanospheres and gold nanoflowers diminished hsp70 levels and O-GlcNAcylation in nuclei and nucleoli, supporting the idea that GNPs compromise nuclear proteostasis in breast cancer cells. Interestingly, during the recovery from hyperthermia, the nucleolar and nuclear levels of hsp70 and O-GlcNAcylation were restored in GNP-incubated cells. This may suggest that GNPs, when combined with hyperthermia, elicit severe stress. GNP-treated cells respond to this condition by increasing hsp70s and O-GlcNAcylated proteins in nucleoli and nuclei.

### 3.3 Impact of gold nanoparticles on protein abundance and O-GlcNAylaction

**Figure 5.**
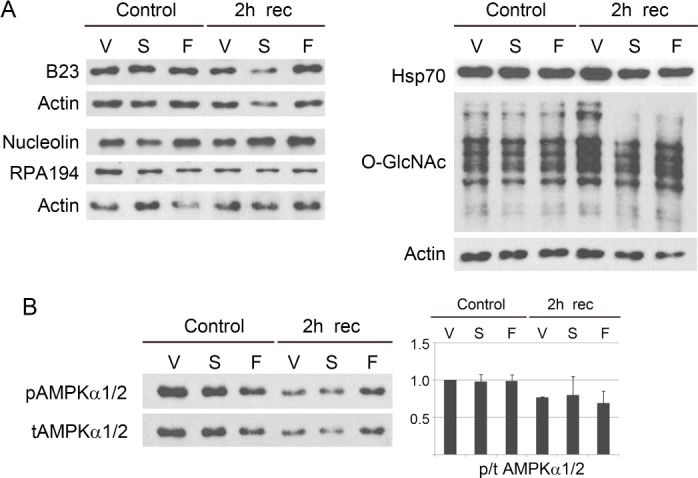
Western blot analysis of nucleolar proteins, hsp70 and AMPK. Crude extracts were prepared for MCF7 cells incubated with vehicle (V), small gold nanospheres (S) or gold nanoflowers (F). Controls were grown at 37°C. Alternatively, cells were exposed to mild hyperthermia followed by 2 hours of recovery. (A) Nucleolar proteins, hsp70, and O-GlcNAcylation were assessed. (B) AMPKα phosphorylation was evaluated; quantification of Western blots was performed for two independent experiments. The results were normalized to controls incubated with vehicle.

Besides their proper distribution, the abundance of nucleolar proteins and molecular chaperones also determines the nucleolar functions and cancer cell survival. Furthermore, the capacity to modify proteins by O-GlcNAcylation indicates the cellular ability to cope with stress. Western blotting was performed to address this point. The most noticeable change for nucleolar proteins was a reduction of B23 in MCF7 cells exposed to small gold nanospheres and hyperthermia (Figure 5A). (Since Figure 1 suggested only minor GNP-dependent effects, and antibodies failed to recognize nucleostemin after blotting, the protein was not included in our analyses).

By contrast, GNPs did not have a pronounced effect on the abundance of nucleolin, RPA194 or hsp70 (Figure 5A). We also did not observe the accumulation of proteolytic fragments for nucleolar proteins in MCF7 cells maintained at 37°C or recovering from heat (not shown). Accordingly, the GNP-induced protein relocation in Figures 1 and 3 did not represent the redistribution of degradation products.

As shown in Figure 5A, the pattern of O-GlcNAcylated proteins was altered in GNP-treated cells during the recovery from hyperthermia. This prompted us to assess 5′-AMP activated protein kinase (AMPK), a stress sensor that plays a complex role in cancer cell metabolism (reviewed in [66]). Relevant to our study, AMPK controls the flux through the hexosamine pathway and thereby regulates O-GlcNAcylation [67]. Moreover, O-GlcNAcylation is critical for the Warburg effect and the survival of different types of breast cancer cells [36]. AMPK also controls the expression of *HSP70* genes [68]. Since GNPs relocated O-GlcNAcylated proteins and hsp70s, we examined whether they altered AMPK activation. To this end, the phosphorylation of AMPKα subunits was measured by Western blotting (Figure 5B). As expected, AMPK activation was diminished during the recovery from heat stress [69]. However, GNPs had no significant effect on AMPKα phosphorylation.

**Figure A3.**
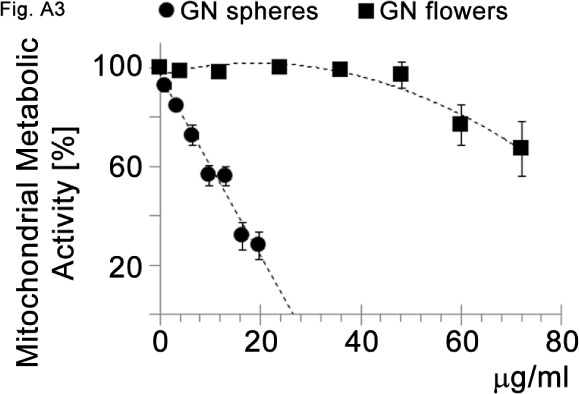
Impact of gold nanoparticles on the metabolic activity of MCF7 cells. The MTT assay assessed the mitochondrial metabolic activity of MCF7 cells incubated with small gold nanospheres (GN spheres) or gold nanoflowers (GN flowers). Data are reproduced from [49] with permission.

We further characterized the impact of GNPs on the metabolic activity of MCF7 cells. As previously shown [49], small gold nanospheres profoundly reduced the mitochondrial activities of MCF7 cells, while gold nanoflowers had a less marked effect (Figure A3). However, due to the higher efficacy of photoconversion into heat, exposure to near-infrared light may enhance cancer cell killing by gold nanoflowers.

## 4. Summary and Perspective

Our current contribution uncovered unique effects of GNPs on MCF7 breast cancer cells. Small gold nanospheres and gold nanoflowers alter the nucleolar and nuclear distribution of B23, RPA194, hsp70 and O-GlcNAc-modified proteins. While B23 [19, 21] and hsp70s [33] are important to cancer cell survival and proliferation, O-GlcNAcylation is essential for breast cancer metabolism [36]. Interestingly, B23 can be immunopurified with antibodies against O-GlcNAc [70], and hsp70s are among the O-GlcNAc-modified proteins [38]. On the other hand, hsp70s interact with B23, and it is conceivable that their posttranslational modifications regulate the B23/hsp70 association. This interaction could be relevant to the role that hsp70s play for the functional integrity of nucleoli, especially upon stress [8, 31].

We have shown GNP-induced changes to nucleolar organization and function in the present and earlier studies [49]. This work provides the foundation for a better mechanistic understanding of GNP-driven events. Our results also support the notion that GNPs modulate two intrinsic cellular cancer support systems: nucleoli and heat shock proteins. We propose that the combination of these effects maximizes cell damage. Identifying the underlying mechanisms and affected components are promising steps towards the ultimate goal of eradicating cancer cells. Based on our results, GNPs not only impinge on nucleoli; they are also potentially useful to alter the pattern and distribution of O-GlcNAcylated proteins. This is relevant because such changes in posttranslational modification could compromise cancer cell proliferation through metabolic reprogramming.

## 5. Conflict of Interest

The authors declare no conflict of interest.
